# Social science contributions to the global action plan on antimicrobial resistance

**DOI:** 10.2471/BLT.25.294438

**Published:** 2025-12-02

**Authors:** Mathieu JP Poirier, Jaskeerat Singh, Isaac Weldon, Clare IR Chandler, Daniela Corno, Laura Valtere, Pedro Henrique D Batista, Daniel Carelli, Geneviève Boily-Larouche, Sonia Lewycka, Fiona Emdin, Kathleen Liddell, Timo Minssen, Ilaria Natali, Susan Nayiga, Iruka N Okeke, Emmanuel Olamijuwon, Kevin Outterson, Julianne Piper, Kayla Strong, Jarnail Singh Thakur, Kednapa Thavorn, Maarten Van Der Heijden, AM Viens, Mary Wiktorowicz, Steven J Hoffman

**Affiliations:** a Global Strategy Lab, York University, 4700 Keele Street, Dahdaleh Building 2120, Toronto, Ontario, M3J 1P3, Canada.; b Center for Advanced Studies in Bioscience Innovation Law, University of Copenhagen, Copenhagen, Denmark.; c Department of Global Health and Development, London School of Hygiene and Tropical Medicine, London, England.; d Max Planck Institute for Innovation and Competition Law, Munich, Germany.; e Department of Technology Management and Economics, Chalmers University of Technology, Gothenburg, Sweden.; f Oxford University Clinical Research Unit, Hanoi, Viet Nam.; g Centre for Law, Medicine and Life Sciences, University of Cambridge, Cambridge, England.; h Toulouse School of Economics, Université Toulouse Capitole, Toulouse, France.; i Department of Pharmaceutical Microbiology, University of Ibadan, Ibadan, Nigeria.; j School of Geography and Sustainable Development, University of St. Andrews, St. Andrews, Scotland.; k Social Innovation on Drug Resistance, Boston University, Boston, United States of America.; l Faculty of Health Sciences, Simon Fraser University, Burnaby, Canada.; m Department of Community Medicine and School of Public Health, Post Graduate Institute of Medical Education and Research, Chandigarh, India.; n Methodological and Implementation Research Program, Ottawa Hospital Research Institute, Ottawa, Canada.; o Dahdaleh Institute for Global Health Research, York University, Toronto, Canada.; Correspondence to Mathieu JP Poirier (email: mathieu.poirier@globalstrategylab.org).

Since the adoption of the *Global action plan on antimicrobial resistance* in 2015,^1^ notable progress has been made in combating antimicrobial resistance. In 2024, 178 countries had developed multisectoral national action plans, with many drawing directly on the global action plan. However, only 20 of these countries have dedicated funding for implementation, 93 have a functioning multisectoral coordinating mechanism and 121 are implementing them.^2^ Furthermore, very few national action plans address inequities in adverse health and social consequences of antimicrobial resistance, including from gender, disability and human rights perspectives.

Current efforts to curb antimicrobial resistance have stalled.^3^ Without faster implementation of effective interventions, including antimicrobial stewardship, water, sanitation and hygiene infrastructure, therapeutics and vaccines, antimicrobial resistance is estimated to reduce global life expectancy by 1.8 years by 2035.^2^

The landscape of global health governance has changed dramatically since the action plan was established. Several cross-border public health crises have occurred, including a pandemic; unprecedented diplomatic challenges for multilateral organizations; and the alteration of the health funding landscape. Nonetheless, public health institutions have strengthened in many parts of the world, and the global health architecture for antimicrobial resistance is maturing through the establishment of institutions such as the Global leaders group on antimicrobial resistance and the AMR multi-stakeholder partnership platform.^2^ The international health community increasingly recognizes that antimicrobial resistance is not only a human, biomedical and regulatory issue, but also a social, animal, ecological and economic one.

Social science research on antimicrobial resistance has gained traction in the last decade, employing a diverse set of theoretical perspectives to better understand topics ranging from antimicrobial stewardship to political coordination.^4^ As the action plan commitments will be updated in 2026, an opportunity exists to employ a broader social science scope to accelerate national antimicrobial resistance interventions.

In January 2025, the Global strategy lab convened leading antimicrobial resistance social scientists from a variety of disciplines to determine which new ways of understanding antimicrobial resistance could catalyse and incentivize action. Three conceptions stood out as important to revisions of the action plan:^2^ antimicrobial resistance as socio-ecological dynamics;^3^ antimicrobials as essential infrastructure;^4^ and antimicrobial resistance as collective action problems.^5^

In this article, we propose that these three social sciences conceptions can be applied to global action plan revisions to improve how problems are defined and their solutions implemented. These three concepts can also engage important new partners to ensure antimicrobial resistance policies are sufficiently equitable, sustainable and multisectoral.

## Socio-ecological dynamics

A socio-ecological lens on antimicrobial resistance emphasizes the omnipresence of microbes in the natural ecology that influence human social relations. Human activities such as antimicrobial use, agriculture, land-use changes, waste disposal and pollution continuously shape microbial environments, which evolve and respond dynamically, creating a feedback loop that affects human health and societal systems.^6^^,^^7^ Agricultural practices play a particularly important role: antimicrobial use in livestock production, crop farming and aquaculture reflect broader economic, social and cultural patterns that, combined, reshape microbial ecologies.

Reframing antimicrobial resistance as an ecological and social sustainability challenge underscores the need to stay within an ecological maximum threshold of global antimicrobial use, while ensuring a minimum standard of equitable access. Staying within an ecological threshold requires responsible antimicrobial use as well as addressing commonly overlooked environmental antimicrobial resistance drivers. One such driver could be air pollution, which recent evidence suggests is correlated with resistance.^7^ This recognition reframes antimicrobial resistance as both a biomedical and environmental health challenge, highlighting the need to include resistance-related benefits in cost–benefit analyses of air quality interventions. Policies that simultaneously address antimicrobial resistance and environmental pollution are likely to be more integrated, efficient and politically appealing.^6^ New regulatory approaches that combine pollution-reduction strategies with antimicrobial resistance mitigation efforts such as clean air standards, low-emission zones and pollution taxes to hold polluters accountable, can help address important underlying environmental drivers of antimicrobial resistance.

A socio-ecological lens highlights that One Health domains and dynamics (human, animal and environmental) shift over time as human needs, technologies and environmental conditions evolve.^8^ Thus, the stakeholders who need to be involved in antimicrobial resistance-mitigation strategies also evolve. This approach promotes collaboration with diverse One Health actors such as those working on air pollution, urban planning and environmental health to unlock additional resources and reduce antimicrobial resistance transmission while advancing shared goals.

## Antimicrobials as infrastructure

Antimicrobial resistance is not only a biomedical issue; a growing body of work reveals how antimicrobials function as essential yet invisible infrastructure underpinning everyday life. Antimicrobials are routinely relied upon in modern food systems, land-use practices, clinical medicine and public health.^8^ However, their critical role, like that of water systems or transportation infrastructure, tends to go unnoticed until failure occurs.

We consider the structural and systemic factors that shape the diverse lived realities dependent on these lifesaving drugs. Most surgical or immunological medical procedures are only safe with effective antimicrobials.^2^ At the same time, marginalized communities rely on antimicrobials to compensate for systemic gaps in health care, sanitation and economic stability.^9^ These challenges are often more pronounced for women, ethnic minorities and migrants, especially in contexts where entrenched inequities increase dependence on antimicrobials and exacerbate antimicrobial resistance risks for all.^9^ Shifting the focus from behavioural to structural drivers of antimicrobial resistance repositions infection prevention as a collective responsibility, and redirects attention from educational correction to equitable support for infection prevention and control.

We also argue that addressing antimicrobial resistance requires sustained public investment and long-term planning, the same as with water and transport systems. Antimicrobials act as a safety net for weak health-care, sanitation and economic systems; hence, this conception strengthens the economic case for long-term investment in mitigating antimicrobial resistance while challenging the notion that heavy reliance on antimicrobials is inevitable. This perspective emphasizes the need to invest in both alternatives and public services to reduce global antimicrobial resistance.

Antimicrobial resistance and antimicrobial use surveillance systems must reveal where and how antimicrobials are being used, and bridge data across sectors and geographies to ensure inclusive and coordinated actions. Doing so includes recognizing the infrastructural role antimicrobials play and enabling locally relevant, targeted interventions.

## Collective action problems 

As a series of collective action problems, antimicrobial resistance is conceptualized as a global social challenge driven by actors with competing interests and limited incentives for international cooperation.^5^ For example, universal and appropriate access to antimicrobials would provide benefits to health security, but efforts to combat antimicrobial resistance have been undermined by resource-constrained health systems, poorly coordinated stakeholders and unaffordable medicines. Poor access to antimicrobials enables disease and resistance to spread: a study estimates that 5.7 million people die every year because of this weakest link.^5^ Consequently, positive externalities of reducing antimicrobial resistance risks are unrealized in the absence of effective treatment options for all. Therefore, individual behaviour or isolated policy changes cannot effectively address antimicrobial resistance. Doing so requires a global action plan that facilitates coordinated, cross-national and systems-wide solutions.

Another commonly cited collective action problem is the ongoing failure to develop new antimicrobials, which is a major challenge to mitigating antimicrobial resistance. New antimicrobials are expensive to develop, and constrained use makes it difficult for companies to recover their costs or make a profit. Consequently, these investments in antimicrobial innovation are not undertaken, many pharmaceutical companies abandon this field, and no widely usable new class of antimicrobials has emerged in decades.^10^

This market failure could be addressed through collective action, including governments’ intervention at the national, regional and multilateral levels. Push mechanisms, such as public research funding, tax credits and public–private partnerships, can directly support antimicrobial resistance research and development. The United Kingdom of Great Britain and Northern Ireland and the European Union have proposed new pull mechanisms such as transferable (data) exclusivity vouchers and antimicrobial products subscription models as complementary solutions.^11^ Sustainable funding for push mechanisms and further conceivable pull mechanisms, such as advanced purchase commitments and patent buyouts, must be prioritized in the revised global action plan for their long-term impacts.^12^

In addition to access and innovation, collective action problems also arise when coordinating stewardship across different jurisdictions and sectors. In Sweden, for example, comparably low antibiotic consumption is largely the result of the Swedish strategic programme against antibiotic resistance. This bottom-up initiative effectively coordinates all hospitals, community health-care centres and political jurisdictions, and is fully integrated into the government’s national action plan.^11^ Coordination challenges are often more pronounced at the transnational level due to differences in national governments’ willingness, capacity and authority to effectively address antimicrobial resistance.^13^

A collective action lens on antimicrobial resistance reveals complex coordination challenges that policy-makers must address. To be effective, the updated action plan must go beyond individual behaviour change and fragmented national efforts to support improved collaboration across sectors and borders. Doing so requires clearly defined responsibilities and a fair distribution of costs, incentives and benefits among the diverse actors involved in antimicrobial resistance governance.

Integrating new social sciences conceptions to coordinate innovation, prevention, access and conservation measures across the complex ecosystem of One Health sectors, actors and countries can greatly strengthen the next iteration of the action plan. Understanding socio-ecological dynamics, antimicrobials as infrastructure and antimicrobial resistance as collective action problems provides a path towards more equitable, sustainable and multisectoral interventions to address the threat of antimicrobial resistance.
